# 58. Discogenic hemivertebral sclerosis mimicking fractures

**DOI:** 10.1093/rap/rky034.021

**Published:** 2018-09-20

**Authors:** Zenib M Sardar

**Affiliations:** Rheumatology, The Royal Wolverhampton Trust, Wolverhampton, UNITED KINGDOM

**Introduction:** Discogenic hemi-vertebral sclerosis is a rare condition of localised sclerotic or lytic lesions affecting vertebral bodies in adults. It is of unknown aetiology and regarded as a do not touch lesion. We report the first case where this lesion has been confused for a fracture.

**Case description:** A 48 year old female patient presented with a 20 year history of mechanical back pain, neck, left shoulder, knee and ankle pain. She had a history of a L5/S1 disc bulge resulting in nerve root compression which was successfully treated with an L5/S1 nerve root block injection under the spinal surgeons three years previously. The patient had a lumbo-sacral radiograph performed due to a back injury 20 years ago which reported multiple Schmorl’s nodes, disc space narrowing at L5/S1 and facetal spondylosis from L4 to S1; namely degenerative changes only. Ongoing back pain led to a repeat lumbar spine radiograph which identified degenerative and modic end plate changes in 2014 and a computed tomography scan of the abdomen and pelvis with contrast 3/11/2014 reported as degenerative changes at L5/S1 does in fact show endplate sclerosis at L5/S1. The patient was never seen by a rheumatologist and no other further testing performed however her father also had a diagnosis of ankylosing spondylitis. Past medical history includes renal calculi, frozen shoulder, double uterus and caesarean section. On examination she was able to mobilise unaided, lumbar spine movements were slightly restricted however Schober’s test was just within normal limits at 5cm. Cervical spine mobility was restricted in extension and rotation at half range movement with crepitus. Left shoulder joint mobility was reduced to flexion 110, abduction 90 and internal rotation ¾ and external rotation half range with a full range of passive movement at the left shoulder. ESR and CRP were not significantly raised at 19 mm/hr (1-12) and 10 mg/l (0-5) respectively, bone profile, Vitamin D studies and immunoglobulins were normal. HLA B27 was negative. MRI of whole spine identified loss of normal cervical lordosis with posterior disc osteophyte complex at C5-C6, C6-C7 and C7-T1 causing thecal sac compression only and minor insignificant disc protrusions were seen at the T-spine. The report stated that in the lumbar spine there is a linear horizontal low signal across the L5 and S1 vertebral body in keeping with a fracture. Short TI inversion recovery identified minor oedema at the site. Other changes include degenerative change and loss of disc height at L5-S1, diffuse disc bulge at L4-5 and mild oedema at left sacroiliac joint inferiorly. These appearances suggest degenerative change. Although the magnetic resonance imaging scan of the spine report suggested the presence of L5 and S1 vertebral body fractures; this seemed unlikely in the absence of any history of trauma and her normal bone mineral density. The dual energy x-ray absorptiometry scan reported a T score of + 1.0 at the femoral neck and +0.6 at the lumbar spine. Therefore further review of the imaging at the rheumatology/radiology meeting with the musculoskeletal radiologist identified discogenic hemi-vertebral sclerosis at the level of L5 and S1 simulating fractures thereby resolving the issue.

**Discussion:** Discogenic hemi-vertebral sclerosis is a rare usually sclerotic, but occasionally lytic or mixed lytic-sclerotic lesion which typically arises in the clinical context of a middle aged woman with chronic low back pain. It has previously been reported that this lesion can be confused with the appearance of a bony metastases or infection but never as a fracture. It is always situated adjacent to the vertebral end plate and usually associated with degenerative changes such as narrowed disc space, Schmorl’s nodes and osteophytes all of which were identified on the patient’s imaging. Discogenic sclerosis can also mimic infectious spondylitis which also presents with narrowed disc space, sclerosis of adjacent vertebral bodies and abnormalities of the vertebral end plate therefore correct identification is vital to prevent unnecessary biopsies and further investigation as the management is conservative. However, diagnosis is challenging and requires correlation of the clinical features with radiological findings which include: rounded sclerosis most frequently in the anterior vertebral body (100%); central lucency in the sclerosis (77%); associated degenerative changes at the intervertebral disc, e.g. vacuum phenomena (44%); absence of a para-spinal mass and maintenance of vertebral body height. This patient underwent several imaging modalities before the correct diagnosis was made. Fortunately, she did not undergo any unnecessary invasive procedures, to repair the supposed vertebral fractures or for further diagnostic value, highlighting the importance of recognising this unusual presentation of discogenic hemi-vertebral sclerosis.

**Key Learning Points:** Discogenic hemi-vertebral sclerosis appears at the end plate and is usually associated with a narrowed disc space and degenerative changes. Discogenic sclerosis can mimic infection, fracture or bony metastases. Correct diagnosis is important to prevent unnecessary biopsy.

**Disclosure: Z.M. Sardar:** None.

